# Mycotoxin Occurrence and Risk Assessment in Gluten-Free Pasta through UHPLC-Q-Exactive Orbitrap MS

**DOI:** 10.3390/toxins13050305

**Published:** 2021-04-25

**Authors:** Josefa Tolosa, Yelko Rodríguez-Carrasco, Giulia Graziani, Anna Gaspari, Emilia Ferrer, Jordi Mañes, Alberto Ritieni

**Affiliations:** 1 Department of Food Chemistry and Toxicology, University of Valencia, Av. Vicent A. Estellés s/n, Burjassot, 46100 Valencia, Spain; emilia.ferrer@uv.es (E.F.); jordi.manes@uv.es (J.M.); 2 Department of Pharmacy, Università di Napoli Federico II, Via D. Montesano, 80131 Napoli, Italy; giulia.graziani@unina.it (G.G.); annagaspari@virgilio.it (A.G.); alberto.ritieni@unina.it (A.R.); 3 UNESCO Chair of Health Education and Sustainable Development at University of Naples, 80131 Napoli, Italy

**Keywords:** mycotoxins, gluten-free pasta, liquid-chromatography, HRMS-Orbitrap, multiresidue method, exposure

## Abstract

Celiac disease (CD) is a genetic-based autoimmune disorder which is characterized by inflammation in the small intestinal mucosa due to the intolerance to gluten. Celiac people should consume products without gluten, which are elaborated mainly with maize or other cereals. Contamination of cereals with mycotoxins, such as fumonisins (FBs) and aflatoxins (AFs) is frequently reported worldwide. Therefore, food ingestion is the main source of mycotoxin exposure. A new analytical method was developed and validated for simultaneous analysis of 21 mycotoxins in gluten-free pasta, commonly consumed by celiac population as an alternative to conventional pasta. Ultrahigh-performance liquid chromatography coupled to quadrupole Orbitrap high-resolution mass spectrometry (UHPLC-Q-Exactive Orbitrap MS) was used for analyte separation and detection. The mycotoxins included in this work were those widely reported to occur in cereal samples, namely, ochratoxin-A (OTA), aflatoxins (AFB1, AFB2, AFG1 and AFG2), zearalenone (ZON), deoxynivalenol (DON), 3-acetyl-deoxynivalenol and 15-acetyl-deoxynivalenol (3-AcDON and 15-AcDON, respectively), nivalenol (NIV), neosolaniol (NEO), fusarenone-X, (FUS-X), T-2 toxin (T-2) and HT-2 toxin (HT-2), fumonisin B1 and B2 (FB1 and FB2, respectively), enniatins (ENN A, ENN A1, ENN B and ENN B1) and beauvericin (BEA). The validated method was successfully applied to 84 gluten-free pasta samples collected from several local markets of Campania region (Italy) during September to November 2020 to monitor the occurrence of mycotoxins and to assess the exposure to these food contaminants. A significant number of samples (95%) showed mycotoxin contamination, being *Fusarium* mycotoxins (FB1, ZON and DON) the most commonly detected ones. Regarding the risk assessment, the higher exposures were obtained for NIV, DON and FB1 for children and teenagers age group which can be explained due to their lower body weight.

## 1. Introduction

Celiac disease (CD), is a serious autoimmune disease occurring in genetically-predisposed people caused by gluten ingestion, a complex of high molecular-weight proteins found in the endosperm of grass-borne grains including wheat, barley and rye [1]. Currently, CD is one of the most common food-induced diseases, with an estimated prevalence ranging from 0.5 to 1% of the population [2]. The treatment of CD is the removal of gluten from the diet (i.e., consumption of a gluten-free (GF) diet) [3]. This is achieved by the consumption of GF foodstuffs, mainly maize, rice, potatoes and other few cereals and pseudo-cereals, which can be safely employed as carbohydrate source since these products do not contain gluten. Apart from celiac population, in recent years, many people prefer to avoid gluten in their diets for fashionable or non-scientific beliefs or because consumers perceive that GF products are healthier. This trend is supported by the increase market of GF products which was valued at 5.6 billion dollars in 2020 and is estimated to reach 8.3 billion dollars in 2025 [4]. Cereals, including those highly consumed in GF diets, are one of the main dietary sources of mycotoxins, and therefore one of the leading foods which increase the exposure to mycotoxins worldwide [5].

Mycotoxins are common contaminants found in cereals and by-products. They can be produced under favorable conditions of temperature and humidity by different filamentous fungi, such as *Aspergillus*, *Penicillium* and *Fusarium*, which can affect crops both in the field and during storage [6]. The most frequently contaminated cereals are wheat, maize, rice, among others. Maize and rice are recognized as good substrates for fungal growth and mycotoxin production, especially aflatoxins (AFs) [7,8], fumonisins (FBs) [9,10], trichothecenes (TCs) [11] and zearalenone (ZON) [12]. Mycotoxins are of significant public health concern, due to their high occurrence and toxic properties [13]. The toxic properties of mycotoxins associated with animals and human beings include carcinogenicity, genotoxicity, teratogenicity, mutagenicity, nephrotoxicity and immunotoxicity. Because of their recognized harmful effects, many countries have adopted regulations to control the mycotoxin exposure [6]. In Europe, maximum residue levels have been set for FBs, AFs, deoxynivalenol (DON), ochratoxin A (OTA), patulin (PAT) and ZON by Commission Regulation 1881/2006/EC [14] and its amendments, setting maximum levels (MLs) for these mycotoxins in maize and maize-based products, which has been modified by Regulation EC 1126/2007 [15], as regards the limits for *Fusarium* mycotoxins in corn and derivatives, based on successive risk assessments and EFSA scientific opinions.

Based on the abovementioned data, the population most exposed to the mycotoxins present in maize and rice foodstuffs (GF products) are celiac people and also some ethnic groups, and within these groups, the highest exposure level corresponds to children. In this sense, the aims of this research were (i) to validate in-house a multi-mycotoxin method for the analysis of 21 different mycotoxins in GF pasta (maize and rice) by ultra-high-performance liquid chromatography coupled to quadrupole Orbitrap mass spectrometry (UHPLC-Q-Orbitrap HRMS) and (ii) to assess the exposure to mycotoxins in celiac population groups through the mycotoxin occurrence data obtained from a surveillance on GF pasta here performed.

## 2. Results and Discussion

### 2.1. Chromatographic and Mass Spectrometric Optimization

The front-end parameters of the Q-Orbitrap, including electrospray ionization (ESI) parameters, the heated capillary and the S-Lens radio frequency (RF) level, were optimized. ESI parameters are responsible for the spraying of the sample carried in the mobile phases followed by either positive or negative ionization modes of the analytes. Those parameters are influenced by both flow rate and mobile phase composition, and hence the mycotoxin standard solutions diluted at 1 µg/mL with mobile phases were individually infused directly into the mass spectrometer through syringe infusion (flow rate: 0.4 mL/min) to mimic working conditions. The flow of sheath gas and auxiliary gas were optimized along with the heater temperature to ensure optimum desolvation of the sample being adjusted to 35 and 10 arbitrary units, respectively. Heater and capillary temperatures were kept at 290 and 305 °C, respectively. The ionization was achieved by applying a spray voltage of 4kV in both ESI modes. Ions are then focused by the S-lens through the application of RF voltage. Different HRMS scan modes were also used (e.g., data-dependent acquisitions) to permit a retrospectively analyze the data if needed for further research purposes [16].

On the other hand, several chromatographic conditions were tested to achieve the best compound separation. Methanol and acetonitrile were tested as organic mobile phase. Both organic solvents showed efficient separation of the studied mycotoxins but methanol was selected due to the superior signal intensity observed. Similarly, ammonium formate (5mM) and formic acid (0.1%) were added as additives to both mobile phases due to enhanced signal intensity. Retention time of the 21 target mycotoxins ranged from 3.83 to 8.24 min with a total run time of 10.0 min. Optimal parameters of studied mycotoxins are presented in Table 1.

### 2.2. Method Validation

The optimized multi-mycotoxin methodology was in-house validated to ensure the reliability of the results. Results are shown in Table 2. The method was found to be selective and specific based on the absence of interference peaks at the retention times of the studied mycotoxins in a blank GF sample (QA/QC sample) injected ten times. Linearity was established in a concentration range from 0.25 to 1000 µg/kg at eight concentration levels in both neat solvent and matrix extract. Calibration standards accuracy of all analytes were within ±15% of the nominal concentrations. Correlation coefficients were greater than 0.990 for all target mycotoxins. Strong to moderate matrix effects were observed (from 38 to 99%) probably due to the simple sample preparation procedure and therefore matrix-matched calibrations were used for quantitation purposes. Spiked samples were used to evaluate trueness and precision of the method. Recoveries ranged between 71 and 125% at all fortification levels (125, 62.5 and 12.5 µg/kg and at 500 µg/kg only for DON and FBs). Repeatability and reproducibility were determined at same spiking levels than trueness and relative standard deviation lower than 10.5 and 12.1% were obtained, respectively. The comprehensive results of accuracy are shown in Table 2. Quality control samples were also included in each batch to guarantee the accuracy of the results. For regulated mycotoxins, all LOD and LOQ were much lower than the maximum level (ML) set in current Regulation [14] and for non-regulated mycotoxins, results were acceptable and comparable to those obtained for regulated mycotoxins. In detail, LODs ranged between 0.05 to 7.8 and LOQs from 0.14 and 23.5 µg/kg.

### 2.3. Mycotoxin Occurrence in Gluten-Free Pasta

Although multi-mycotoxin methods in GF products have been commonly based on the detection of AFs, FBs, and ZON, in the present study, the determination of up to 21 mycotoxins in GF pasta samples has been carried out by in house method validation, thus supposing an improvement for the field of mycotoxin determination on GF products. Moreover, as our knowledge, multi-mycotoxin determination in GF pasta by Orbitrap mass spectrometry have not been reported to date.

The validated method was successfully applied for the screening of mycotoxins in eighty-four commercially available GF pasta commodities. The mycotoxin occurrence and their levels obtained in the present study are reported in Table 3. As it can be observed, ninety-five percent of GF pasta samples included in the survey shown to be contaminated by one or more than one mycotoxin, being common the mycotoxin co-occurrence of more than one mycotoxin simultaneously. Only one sample showed no mycotoxin contamination (a parboiled Italian rice sample).

Regarding mycotoxin occurrence, the most prevalent mycotoxins found in analyzed samples were those belonging from *Fusarium* genera. The incidence of contamination was FB1 > ZON > DON > NIV = FB2. In detail, the highest percentages of occurrence corresponded to FB1, ZON and DON, with an overall incidence of 90.5%, 71.4% and 66.7%, respectively. On the other hand, regarding mycotoxin contents, the order of mycotoxin levels was DON > NIV > FB1 > FB2 > ZON > HT-2. The highest contents corresponded to DON (377.4 µg/kg), followed by NIV (367.6 µg/kg). The highest DON content (377.4 µg/kg) corresponded to a sample of “pasta dietetica” (fusilli) consisting in rice flour (67%) and corn flour (33%). In this sample, DON was found simultaneously with NIV (211.7 µg/kg), ZON (10.4 µg/kg) and FB1 (39.9 µg/kg). A 100% buckwheat pasta sample showed the highest NIV content (367.6 µg/kg) in co-occurrence with DON (240 µg/kg), HT-2 toxin (11.3 µg/kg), and ZON (26.3 µg/kg). Although mycotoxin contents detected were high, especially for DON, NIV and FBs, none of the samples exceed the MLs established by the European Regulation [14].

The co-occurrence of two or more mycotoxins has been commonly reported in analyzed samples. In this sense, only the 9% of commodities were contaminated by one mycotoxin. The 11% of samples were contaminated simultaneously by two mycotoxins, being the most common combination DON with FB1 and ZON with FB1. The combination of three mycotoxins with (NIV, DON and FB1 and DON, ZON and FB1) were the most commonly detected (44% of samples). The 26% of samples showed the presence of four mycotoxins simultaneously, being DON, ZON, NIV and FBs in different combinations the most frequently detected. Finally, 4% of samples were contaminated by five mycotoxins and 5% of samples presented co-occurrence of up to six mycotoxins. These results suggest that special attention should be paid to samples showing the presence of more than one mycotoxin simultaneously, as their toxic effects produced by their interaction could be synergistic.

Emerging *Fusarium* mycotoxins have been extensively reported to occur in cereal samples even at high contents [17], however, ENN A and ENN B were not detected in GF pasta samples analyzed in the present study, and lower incidence and contents were detected for ENN A1 and BEA (4.8% and 9.5% at 1.7 µg/kg and 19.6 µg/kg, respectively). Regarding mycotoxins *from Aspergillus* and *Penicillium* genera included in the study (AFs and OTA, respectively), no mycotoxin contents were detected in GF samples analyzed.

With regard to mycotoxin occurrence in pasta samples, differences have been observed between GF pasta and durum wheat pasta in relation to mycotoxin contamination, as different ingredients are used for the elaboration and some matrices are more prone to contamination by certain mycotoxins than others. Thus, whereas FBs, mainly FB1 and FB2, were the most detected mycotoxins in the present study in GF pasta, no FB contents were detected in a previous survey on wheat-based pasta commodities [17]. DON and ZON were the predominant mycotoxins in durum wheat pasta (100% and 93%, respectively); however, although higher incidence was reported in these samples, higher contents were reported for DON in GF pasta (239.8 µg/kg in GF pasta vs. 96.9 µg/kg in durum wheat pasta). On the other hand, ENNs showed higher incidence and contents in durum wheat pasta in contrast to GF pasta. ENN B was detected in 90% of durum wheat pasta with contents up to 710.9 µg/kg; however, ENN B was not detected in GF pasta samples [17].

Mycotoxin contents detected in GF pasta samples surveyed in this study was in accordance with data reported by other authors. The detected mycotoxins have been previously reported in different GF samples [18,19], especially FBs, which were the most analyzed and reported mycotoxins in GF foodstuffs. In the study conducted by Esposito et al. (2016) [18], 154 Italian GF products (breakfast cereals, biscuits, bread, canned corn, cornmeal, rice, pasta, cookies, sweet and savory snacks) were analyzed, showing an incidence of 85% for FBs (FB1, FB2 and FB3), with levels up to 272 µg/kg. Furthermore, high FBs levels were found in maize, corn-meal, and maize-flour samples in the survey conducted by Magro et al. (2011) [19], where 7% of analyzed samples showed contents above the ML fixed by the EU Regulation 1126/2007/EC [15]. FBs were also reported by Dall’Asta et al. (2009) [20] in 90% of the GF food samples reaching a maximum concentration level of 3310 µg/kg. Moreover, in many cases the sum of free and bound FBs exceeded the EU legal limit set for total FBs. Dall’Asta et al. [21] reported an incidence of 89% of FBs in GF products intended for celiac population and 7% of the samples exceed the EU legal limits [14]. Regarding pasta samples, these authors found a 93% of incidence and contents ranged from 27 µg/kg to 335 µg/kg. Results reported by Cano-Sancho et al. [22] showed the co-occurrence of FB1 and FB2 in most of the analyzed GF commodities together with AFs, DON and ZON. Our results were in accordance with those FBs contents reported by the above-mentioned studies, where FB1 showed higher incidence than FB2. Other studies have also reported high FBs (FB1 and FB2) contents [6,23], with maximum levels of 421 and 759 µg/kg, respectively. Furthermore, Huong et al. [24] found that 24% of maize samples and 8% of rice analyzed in their study were contaminated by FBs with contents ranging from 5.6 to 89.8 µg/kg and from 2.3 to 624 µg/kg, respectively.

Regarding DON occurrence, Cano Sancho et al. [22] reported DON levels only in two samples out of 18 GF pasta analyzed, with respective amounts of 163 and 270 µg/kg. In the survey performed by Herrera et al. [25], DON was investigated in 27 samples of GF samples intended for children age between 4 to 6 months, showing an incidence of 22% of positive samples with contents ranging from 33 µg/kg to 194 µg/kg. As regards the presence of ZON in GF samples, Cano Sancho et al. [22] reported ZON contamination in 6 ethnic food samples but ZON was not detected in any GF sample, which was in accordance with results reported by Brera et al. [6] where no ZON contamination was found in GF pasta samples. Contrary to these surveys, ZON showed a high incidence in GF pasta analyzed in our study.

Regarding emerging *Fusarium* mycotoxin levels, different pattern of contamination was observed. In the present study, only ENN A1 and BEA were detected in one and two samples, respectively. These findings agree with those reported by Decleer et al. [26], where trace levels of ENN A1 were reported. However, these authors reported ENN B and ENN B1 as the most prevalent mycotoxins, while ENNs type B were not detected in our study. In addition, BEA levels found by these authors were higher (up to 209.0 ± 39.7 µg/kg) than those obtained in our study.

Regarding AFs contamination, although AFs have been widely reported especially in maize-based foodstuffs, AFB1 was not detected in the present survey, according to studies carried out by Brera et al. [6] and Cano Sancho et al. [22], where AFB1 was not detected in any sample. On the other hand, other mycotoxins such as OTA and T-2 toxins were not detected in our study, whereas Brera et al. [6] found contents of these two mycotoxins in analyzed samples with some samples exceeding the maximum level set for OTA by the European legislation (Commission Regulation 1881/2006) [14].

### 2.4. Exposure Assessment

To evaluate the mycotoxin exposure through the diet for CD patients, contents detected in the present survey were combined with data consumption of Italian population. Table 4 summarizes the exposure assessment of mycotoxins evaluated in different population groups based on pasta consumption of Italian population. The results were calculated following the equation described in Section 4.7. The Probable Daily Intake (PDI) was calculated only for detected mycotoxins, including NIV, DON, HT-2, ZON, FB1, FB2, ENN A1 and BEA. The PDI have been calculated by using the mean consumption value for the total population consulted for each age group and, as a worst-case scenario, exposure was calculated at 95th percentile of consumption. The PDI for age group from 0 to 2.9 years was not calculated since this age group are not regular consumers of the products included in the study. As reported in Table 4, the PDI for all detected mycotoxins were below the corresponding TDI established; however, the TDI levels for children (3–9.9 years) were surpassed for NIV, DON and the sum of T-2 and HT-2 toxins when assumed the 95th percentile of consumption. It must be highlighted that for all mycotoxins children age group (3–9.9 years) showed the highest PDI among all the age groups although the mean consumption and the P95 consumption were lower than that reported for other age groups. This can be explained by their lower body weight and thus, the unfavorable body weight/intake ratio, which result in a higher exposure [6].

The results reported in Table 4 suggest that mycotoxin exposure level might not pose a health risk for the average consumers; however, for certain populations, especially infants or children age group, and for heavy consumers, mycotoxin intake with GF pasta samples could exceed the safety limits. In this sense, although the common GF foodstuffs available for consumers are mainly intended for adults, children can be common consumers of this kind of products because CD is often diagnosed in early ages. For this reason, mycotoxin limits established for maize-based products intended for children should be considered, as they are lower than those established for adult population.

In addition, some studies revealed that celiac population may have important health implications consuming GF products due to the high occurrence of FBs in these products which implies a high FBs intake for celiac patients [21]. Nevertheless, in this study, PDI calculated for FBs (FB1 and FB2) did not exceed the TDI established for the sum of FBs (Table 4). On the other hand, PDI calculated for emerging *Fusarium* mycotoxins could not be compared to the limits established as no TDI has been established nor for ENNs neither for BEA.

## 3. Conclusions

An UHPLC-Q Exactive Orbitrap MS method has been in-house validated for the simultaneous determination of 21 mycotoxins. Results showed that the proposed analytical procedure was accurate (recovery range from 71 to 125% for vast majority of analytes), precise (RSDs < 12.1%), and sensitive (LODs from 0.05 to 7.8 μg/kg) to fulfil the criteria established in European guidelines. FBs, ZON and DON were found as common contaminants in maize-based foodstuffs, underlining that these foodstuffs can be contaminated with *Fusarium* mycotoxins at levels that could represent a risk for the higher consumers of these products, especially celiac people, due to the continuous ingestion of these foodstuffs. Regarding exposure assessment, it has been determined that GF consumers are exposed to mycotoxin ingestion, and exposure to mycotoxins could suppose a risk especially in the worst-case scenario for higher consumers and concretely for children age group. Thus, with the aim of reducing the risk associated to the ingestion of mycotoxins commonly present in these foodstuffs, research on mycotoxin contamination in these products and their mitigation should be carried out.

## 4. Materials and Methods

### 4.1. Chemicals and Reagents

Mycotoxin standards, namely aflatoxins (AFB1, AFB2, AFG1 and AFG2) fumonisins (FB1 and FB2), ochratoxin A (OTA), zearalenone (ZON), enniatins (ENN A, ENN A1, ENN B and ENN B1), beauvericin (BEA), deoxynivalenol (DON), 3-acetyldeoxynivalenol (3-ADON), 15-acetyldeoxynivalenol (15-ADON), nivalenol (NIV), T-2 toxin (T-2), HT-2 toxin (HT-2), neosolaniol (NEO), and fusarenon-X (FUS-X), with purity ≥98%, were obtained from Sigma Aldrich (Milan, Italy) and they were stored at -20°C. Acetonitrile (MeCN), methanol (MeOH), and water were LC-MS grade and acquired from Merck (Darmstadt, Germany). Formic acid and ammonium formate were supplied by Fluka (Milan, Italy), whereas magnesium sulphate and sodium chloride were purchased from Sigma Aldrich (Milan, Italy).

Syringe filters with politetrafluoroethylene membrane (PTFE, 15 mm, diameter 0.2 mm) were purchased from Phenomenex (Castel Maggiore, Italy). Centrifuge polypropylene tubes Corning PQ of 50 and 15 mL were provided by Corning Cable Systems (SRL, Turin, Italy).

Standard stock solutions of individual mycotoxins were prepared dissolving them in MeCN to reach a final concentration of 1 mg/mL, except for FB1 and FB2, which were prepared in MeCN/H_2_O 50:50 *v/v* solution. Subsequently, working standard solutions containing all the investigated analytes were prepared at appropriate concentration levels to conduct spiking experiments. All stock and working standards solutions were stored in safety conditions at −20 °C.

### 4.2. Sampling

Eighty-four GF pasta samples were evaluated to perform a surveillance of mycotoxins. Commercially available samples were acquired from several local markets of Campania region (Italy) during September to November 2020. This region was chosen due to the incidence of CD per 100,000 person-years amounted to 7.3 and 27.4 for adults and children, respectively, representing the third region for CD frequency in Italy [28].

As far as sample composition is concerned, all of collected samples contained maize and rice flour in different proportions (generally 30:70 *w/w*, respectively), with the exception of one 100% rice sample, one 100% buckwheat sample and two 100% maize flour samples. According to Commission Regulation EC/401/2006 [29], samples were milled throughout a high-speed food blender (Ika, mod. A11 basic, Staufen, Germany) and stored in a dark and dry place at 4 °C until analysis.

### 4.3. Sample Preparation

Sample preparation procedure reported by Rodríguez-Carrasco et al. (2014) [11] was followed and slightly modified. Briefly, 4 g of ground sample were weighted in a 50 mL centrifuge tube and 7.5 mL of water with 0.1% (*v/v*) formic acid and 10 mL of MeCN were added. Then, the mixture was vortexed for 3 min and then 1 g of NaCl and 4 g of MgSO_4_ were added. After that, the mixture was vortexed for 2 min and centrifuged at 5000 rpm (1960× *g*) for 5 min and a 0.5 mL of supernatant was diluted with deionized water in 1:1 (*v/v*) ratio. Finally, the extract of sample was filtered through the 0.2 µm filter and transferred to an autosampler vial for the UHPLC-Q-Orbritrap HRMS analysis.

### 4.4. Ultra-High Performance Liquid Chromatography Couple to Q Exactive Orbitrap Mass Spectrometry (UHPLC-Q-Orbritrap HRMS) Analysis

An Ultra-High-Performance Liquid Chromatograph (UHPLC, Thermo Fisher Scientific, Waltham, MA, USA) equipped with a degassing system, a Dionex Ultimate 3000 Quaternary UHPLC pump working at 1250 bar, an autosampler device and a thermostated (40 °C) Luna Omega C18 (50 × 2.1 mm, 1.6 µm particle size) column (Phenomenex, Castel Maggiore, Italy) were used to obtain the qualitative and quantitative profile of mycotoxins. The mobile phase composition consisted of (A) H_2_O in 0.1% formic acid and 5 mM ammonium formate, and (B) MeOH in 0.1% formic acid and 5 mM ammonium formate. Mycotoxins were eluted using a 0.4 mL/min flow rate with the following gradient elution program: 0–0.5 min 20% of phase B, 1 min 40% of phase B, 6 min 100% of phase B, 8 min 20% of phase B, 10 min 20% of phase B. Injection volume was of 1 μL.

Detection was performed using a Q-Exactive Orbitrap mass spectrometer (Thermo Fisher Scientific). Full scan data in both positive and negative mode were acquired at resolving power of 70,000 FWHM at m/z 200. Ion source parameters in negative mode (ESI-) were: spray voltage −4kV, sheath gas (N_2_ > 95%) 35, auxiliary gas (N_2_ > 95%) 10, capillary temperature 290 °C, S-lens RF level 50, auxiliary gas heater temperature 305 °C. Ion source parameters in positive mode (ESI+) were: spray voltage 4kV, sheath gas (N_2_ > 95%) 35, auxiliary gas (N_2_ > 95%) 10, capillary temperature 290 °C, S-lens RF level 50, auxiliary gas heater temperature 305 °C. A scan range of m/z 100–1000 was selected. The automatic gain control (AGC) was set at 1 × 10^6^ and the injection time was set at 200 ms. Scan rate was set at 2 scans/s [30]. The accuracy and calibration of the HRMS instrument was tested daily using the reference standard mixture obtained from Thermo Fisher Scientific. Data processing was performed using the Xcalibur software, v. 3.0.63 used in 2020 (Xcalibur, Thermo Fisher Scientific, Waltham, MA, USA).

### 4.5. Method Performance

The proposed UHPLC-Q-Orbitrap HRMS method was in-house validated in terms of linearity, trueness, repeatability, within-laboratory reproducibility, limit of detection (LOD) and limit of quantitation (LOQ), according to the EU Commission Decision 2002/657/EC [31]. Linearity, expressed as correlation coefficient, was assessed by constructing calibration curves for all mycotoxins ranging from 0.25 to 1000 µg/kg. The data were fit to a linear least-squares regression curve with a 1/x weighting, and they were not forced through the origin. Linearity was assumed when regression coefficients were greater than 0.990 with residuals lower than 30%. To assess the matrix effect on the chromatographic response, the slopes of the calibration in standard solution with those obtained in matrix-matched standards were compared and expressed as percentage of signal suppression/enhancement (% SSE), as follows:

(1) $$\%\mathrm{SSE}=\left(1-\frac{Sm}{Ss}\right)\times 100$$

where *Sm* is the slope of calibration curve in matrix-matched standard and *Ss* is the slope of calibration curve in standard solution.

The trueness was evaluated throughout recovery studies. Recovery assays were performed in triplicate at three fortification levels (125, 62.5 and 12.5 µg/kg). An additional spiking level of 500 µg/kg was included for DON and FBs due to the higher permitted maximum limits of these mycotoxins in cereal-based foodstuffs. Spiked samples were placed overnight, and then the samples were extracted as previously mentioned. The precision of the method was calculated by the repeated analysis of spiked samples at all tested fortification levels and expressed as the relative standard deviation (%RSD) of measurements (*n* = 3) carried out in the same day (repeatability) and in three different days (within-laboratory reproducibility). LOD was established as the minimum concentration where the molecular ion can be identified (mass error value below 5 ppm) and LOQ was set as the lowest concentration that allowed the concentration of the analyte to be determined with accuracy and precision ≤20%.

### 4.6. Quality Assurance/Quality Control (QA/QC)

Chromatographic and mass spectrometry data were used for confirmation. In detail, mycotoxin identification was carried out by two identification points: the peaks for the mycotoxins included in the study were confirmed by comparing the retention time from extracted ion chromatogram (XIC) of the peak in samples with those of standard solutions at a tolerance of ± 2.5%; and by the exact mass set to five decimal places. Mycotoxin standards chromatograms are provided in Supplementary material (Figure S1). The mass accuracy (∆) for a measured ion was calculated according to the following formula and expressed as part-per-million (ppm) [17]:

(2) $$\Delta \left(\mathrm{ppm}\right)=1\times 10^{6}\frac{\left(m/z_{\mathrm{measured}}-m/z_{\mathrm{theoretical}}\right)}{m/z_{\mathrm{theoretical}}}$$

In order to demonstrate the effectiveness of the validated method, a reagent blank, a blank sample, a replicate sample, and a matrix-matched calibration were included at the beginning and at the end of each batch of samples for QA/QC analysis. Samples were analyzed in duplicate; measurable concentrations of mycotoxins were detected in both replicates.

### 4.7. Mycotoxin Probable Daily Intakes Calculation

The exposure assessment was carried out based on a deterministic approach by combining the mean content of a mycotoxin *Cm* (µg/kg) obtained from the samples here analyzed and the food consumption data *K* (g/day) consulted in the survey published by the Italian Institute of Nutrition (INRAN) [28] of different population groups: children (3–9.9 years), teenagers (10–17.9 years), adults (18–64.9 years), and elderly (65 years and above). Starting from the teenagers age group, food consumption was also differentiated per gender. And body weight data were assumed those reported in the INRAN survey for the defined population groups. Thus, the probable daily intake (PDI, µg/kg bw/day) of each mycotoxin *m*, was assessed as follows [32]:

(3) $${\mathrm{PDI}}_{m}=\frac{C_{m}\times K}{\mathrm{bw}}$$

Additionally, a worst-case scenario was also considered by taking into account the 95th percentile of food consumption data to assess the exposure for those large scale consumers.

## Figures and Tables

**Table 1:** UHPLC/ESI Q-Orbitrap optimized parameters of analyzed mycotoxins.

Mycotoxin	Retention Time (min)	Elemental Composition	Adduct Ion	Theoretical Mass (m/z)	Measured Mass (m/z)	Accuracy (Δ ppm)
3-AcDON	3.83	C_17_H_22_O_7_	[M + H]^+^	339.14383	339.14331	−1.53
15-AcDON	4.02	C_17_H_22_O_7_	[M + H]^+^	339.14383	339.14331	−1.53
DON	4.18	C_15_H_20_O_6_	[M + HCOOH]^−^	341.12451	341.12454	0.09
NIV	4.35	C_15_H_20_O_7_	[M + H]^+^	313.12810	313.12785	−0.74
FUS-X	4.47	C_17_H_22_O_8_	[M + H]^+^	355.13874	355.13866	−0.92
NEO	4.58	C_19_H_26_O_8_	[M + NH_4_]^+^	400.19659	400.19653	−0.15
AFG2	4.61	C_17_H_14_O_7_	[M + H]^+^	331.08123	331.08032	−2.75
AFG1	4.79	C_17_H_12_O_7_	[M + H]^+^	329.06553	329.06553	−0.05
AFB2	4.98	C_17_H_14_O_6_	[M + H]^+^	315.08631	315.08521	−3.49
AFB1	5.02	C_17_H_12_O_6_	[M + H]^+^	313.07066	313.06958	−3.45
HT-2	5.63	C_22_H_32_O_8_	[M + NH_4_]^+^	442.24354	442.24323	−0.70
FB1	6.03	C_34_H_59_NO_15_	[M + H]^+^	722.39575	722.39539	−0.50
T-2	6.13	C_24_H_34_O_9_	[M + NH_4_]^+^	484.25411	484.25418	0.14
OTA	6.50	C_20_H_18_NO_6_Cl	[M + H]^+^	404.08954	404.08801	−3.79
ZON	6.53	C_18_H_22_O_5_	[M + H]^+^	317.13945	317.13910	−1.10
FB2	6.78	C_34_H_59_NO_14_	[M + H]^+^	706.40083	706.40192	1.54
ENN B	7.81	C_33_H_57_N_3_O_9_	[M + NH_4_]^+^	657.44331	657.44299	−0.49
BEA	7.96	C_45_H_57_N_3_O_9_	[M + NH_4_]^+^	801.44330	801.44323	−0.09
ENN B1	8.06	C_34_H_59_N_3_O_9_	[M + NH_4_]^+^	671.45986	671.45923	−0.94
ENN A1	8.11	C_35_H_61_N_3_O_9_	[M + NH_4_]^+^	685.47461	685.47351	−1.60
ENN A	8.24	C_36_H_63_N_3_O_9_	[M + NH_4_]^+^	699.49026	699.48926	−1.43

**Table 2:** Analytical parameters (recovery at spiking level of 12.5, 62.5 and 125 µg/kg and 500 µg/kg for deoxynivalenol and fumonisins, intraday and interday precision, signal suppression enhancement (SSE), limits of detection and quantitation (LOD and LOQ, respectively), calibration curves and correlation coefficient (*r*^2^) for each mycotoxin in analyzed samples).

Mycotoxin	Recovery (%)			Repeatability RSD %	Reproducibility RSD %	SSE (%)	LOD (µg/kg)	LOQ (µg/kg)	Calibration Curves	*r* ^2^
	12.5 µg/kg	62.5 µg/kg	125 (500 *) µg/kg							
3-AcDON	71	92	83	10.2	10.5	73	1.1	3.3	*y* = 282,673*x*	0.992
15-AcDON	70	94	82	9.9	10.1	75	1.1	3.3	*y* = −382,359 + 25,694.5*x*	0.992
DON	89	85	98 (95 *)	5.8	6.7	94	2.1	6.4	*y* = −451,745 + 27,781.9*x*	0.997
NIV	68	79	75	6.2	5.6	75	5.4	16.1	*y* = −558,243 + 37,636.8*x*	0.996
FUS-X	97	110	90	10.5	10.1	48	7.8	23.5	*y* = −593,326 + 10,261*x*	0.997
NEO	114	118	122	5.2	4.0	39	2.3	6.9	*y* = −1.20903 × 10^6^ + 275,818*x*	0.997
AFG2	102	112	98	6.5	7.2	78	0.10	0.23	*y* = 71,914 + 3.10136 × 10^6^*x*	0.996
AFG1	95	110	102	6.3	7.5	84	0.10	0.23	*y* = 68,876 + 3.09432 × 10^6^*x*	0.997
AFB2	105	125	120	8.4	10.4	80	0.06	0.17	*y* = 71,914.2 + 3.13427 × 10^6^*x*	0.992
AFB1	121	120	124	1.8	2.1	83	0.06	0.17	*y* = 72,965.1 + 3.08148 × 10^6^*x*	0.994
HT-2 toxin	112	95	98	8.7	9.1	87	1.2	3.6	*y* = −146,011 + 186,971*x*	0.997
FB1	104	115	99 (104 *)	8.3	8.2	72	1.9	5.6	*y* = −285,447 + 74,040.9*x*	0.998
T-2 toxin	121	107	98	9.4	11.6	66	0.8	2.5	*y* = −1.7152 × 10^6^ + 1.58156 × 10^6^*x*	0.999
OTA	95	100	118	10.3	12.1	91	0.08	0.25	*y* = −40,0195 + 21,4596*x*	0.998
ZON	90	93	105	8.7	7.9	87	0.13	0.38	*y* = 1.37666 × 10^6^ + 937,950*x*	0.997
FB2	99	110	115 (108 *)	7.1	8.2	85	0.26	0.76	*y* = −414,600 + 89,251.2*x*	0.998
ENN B	90	108	117	7.4	10.7	41	0.05	0.16	*y* = −497,411 + 82,621.5*x*	0.990
BEA	99	96	112	6.2	8.5	88	3.6	10.9	*y* = −272,121 + 58,199.7*x*	0.995
ENN B1	102	123	120	7.8	11.4	38	0.05	0.76	*y* = −435,829 + 78,581*x*	0.992
ENN A1	113	110	109	3.5	2.1	86	0.10	0.30	*y* = −476,813 + 79,612.5*x*	0.996
ENN A	123	118	115	3.7	4.0	99	0.05	0.14	*y* = −387,123 + 68,591.5*x*	0.998

* Additional fortification level (500 µg/kg) for deoxynivalenol (DON) and fumonisins (FB1 and FB2).

**Table 3:** Incidence and mycotoxin contents in samples analyzed, IARC classification and MLs established for cereal foodstuffs.

Mycotoxin	Incidence (%)	Range (Mean) (µg/kg)	IARC Classification	MLs (EC) No. 1881/2006 (µg/kg)
NIV	33.3	209.2–367.6 (241.3)	3	No limits established
DON	66.7	182.2–377.4 (239.8)	3	750
HT-2 toxin	9.5	18.2–26.3 (22.2)	NC	No limits established
ZON	71.4	9.2–26.9 (13.5)	3	20–200 ***
FB1	90.5	39.9–246.9 (116.2)	2B	200 **–400
FB2	33.3	44.0–53.4 (48.0)	2B	
ENN A1	4.8	1.7 *	NC	No limits established
BEA	9.5	17.3–21.9 (19.6)	NC	No limits established

*: only one positive sample; **: products intended for children; ***: maize-based products; NC: not classified.

**Table 4:** Risk characterization based on Exposure assessment of mycotoxins studied in different population groups for pasta. The results are reported per age/sex category; overall exposures are reported.

Mycotoxin	Age/Sex (Years)	Bodyweight (kg) *	Mean Consumption (g) *	Consumption P95 (g) *	Contamination Range (µg/kg)	Exposure (ng/kg bw/day)	Exposure P95 (ng/kg bw/day)	TDI (ng/kg bw/day)
NIV	3–9.9 M/F	26.1	58.2	104.9	209.2–367.6	466.5–819.7	840.8–1477.4	1200 (SCF, 2013)
	10–17.9 M	57.1	63.6	128		233.0–409.4	468.9–824.0	
	10–17.9 F	49.1	56.6	105.3		241.1–423.7	448.6–788.3	
	18–64.9 M	78.4	60.3	118.4		160.9–282.7	315.9–555.1	
	18–64.9 F	62.2	47.4	100		159.4–280.1	336.3–590.9	
	≥65 M	78.1	61.1	109.6		163.6–287.6	293.6–515.9	
	≥65 F	65	50.7	100.6		163.2–286.7	323.8–569.0	
DON	3–9.9 M/F	26.1	58.2	104.9	182.2–377.4	406.3–841.5	732.3–1516.8	1000 (SCF, 2004)
	10–17.9 M	57.1	63.6	128		202.9–420.4	408.4–846.0	
	10–17.9 F	49.1	56.6	105.3		210.0–435.0	390.7–809.4	
	18–64.9 M	78.4	60.3	118.4		140.1–290.3	275.1–570.0	
	18–64.9 F	62.2	47.4	100		138.8–287.6	292.9–606.7	
	≥65 M	78.1	61.1	109.6		142.5–295.2	255.7–529.6	
	≥65 F	65	50.7	100.6		142.1–294.4	282.0–584.1	
HT-2 toxin	3–9.9 M/F	26.1	58.2	104.9	18.2–26.3	40.6–58.6	73.1–105.7	Σ T-2 + HT-2 100 (SCF, 2011a)
	10–17.9 M	57.1	63.6	128		20.3–29.3	40.8–59.0	
	10–17.9 F	49.1	56.6	105.3		21.0–30.3	39.0–56.4	
	18–64.9 M	78.4	60.3	118.4		14.0–20.2	27.5–39.7	
	18–64.9 F	62.2	47.4	100		13.9-20.0	29.3–42.3	
	≥65 M	78.1	61.1	109.6		14.2–20.6	25.5–37.0	
	≥65 F	65	50.7	100.6		14.2–20.5	28.2–40.7	
ZON	3–9.9 M/F	26.1	58.2	104.9	9.2–26.9	20.5–60.0	37.0–108.1	250 (SCF, 2011b)
	10–17.9 M	57.1	63.6	128		10.2–30.0	20.6–60.3	
	10–17.9 F	49.1	56.6	105.3		10.6–31.0	19.7–57.7	
	18–64.9 M	78.4	60.3	118.4		7.1–20.7	13.9–40.6	
	18–64.9 F	62.2	47.4	100		7.0–20.5	14.8–43.2	
	≥65 M	78.1	61.1	109.6		7.2–21.0	12.9–37.7	
	≥65 F	65	50.7	100.6		7.2–21.0	14.2–41.6	
FB1	3–9.9 M/F	26.1	58.2	104.9	39.9–246.9	89.0–550.6	160.4–992.3	Σ FB1 + FB2 2000 (SCF, 2003)
	10–17.9 M	57.1	63.6	128		44.4–275.0	89.4–553.5	
	10–17.9 F	49.1	56.6	105.3		46.0–284.6	85.6–529.5	
	18–64.9 M	78.4	60.3	118.4		30.7–189.9	60.3–372.9	
	18–64.9 F	62.2	47.4	100		30.4–188.2	64.1–397.0	
	≥65 M	78.1	61.1	109.6		31.2–193.2	56.0–346.5	
	≥65 F	65	50.7	100.6		31.1–192.6	61.8–382.1	
FB2	3–9.9 M/F	26.1	58.2	104.9	44.0–53.4	98.1–119.1	176.8–214.6	Σ FB1 + FB2 2000
	10–17.9 M	57.1	63.6	128		49.0–59.5	98.6–119.7	
	10–17.9 F	49.1	56.6	105.3		50.7–61.6	94.4–-114.5	
	18–64.9 M	78.4	60.3	118.4		33.8–41.1	66.4–80.6	
	18–64.9 F	62.2	47.4	100		33.5–40.7	70.7–85.9	
	≥65 M	78.1	61.1	109.6		34.4–41.8	61.7–74.9	
	≥65 F	65	50.7	100.6		34.3–41.7	68.1–82.6	
ENN A1	3–9.9 M/F	26.1	58.2	104.9	1.7	3.8	6.8	No TDI established
	10–17.9 M	57.1	63.6	128		1.9	3.8	
	10–17.9 F	49.1	56.6	105.3		1.9	3.6	
	18–64.9 M	78.4	60.3	118.4		1.3	2.6	
	18–64.9 F	62.2	47.4	100		1.3	2.7	
	≥65 M	78.1	61.1	109.6		1.3	2.4	
	≥65 F	65	50.7	100.6		1.3	2.6	
BEA	3–9.9 M/F	26.1	58.2	104.9	17.3–21.9	38.6–48.8	69.5–88.0	No TDI established
	10–17.9 M	57.1	63.6	128		19.3–24.4	38.8–49.1	
	10–17.9 F	49.1	56.6	105.3		19.9–25.2	37.1–47.0	
	18–64.9 M	78.4	60.3	118.4		13.3–16.8	26.1–33.1	
	18–64.9 F	62.2	47.4	100		13.2–16.7	27.8–35.2	
	≥65 M	78.1	61.1	109.6		13.5–17.1	24.3–30.7	
	≥65 F	65	50.7	100.6		13.5–17.1	26.8–33.9	

*: Data reported by Leclerq et al. [27]. M: male; F: female.

## Data Availability

The data presented in this study are available on request from the corresponding author.

## Supplementary Materials

The following are available online at https://www.mdpi.com/article/10.3390/toxins13050305/s1, Figure S1: UPLC-Q-Orbitrap HRMS extracted ion chromatogram of a blank GF sample spiked at 12.5 µg/kg of each mycotoxin.
