# Supplementary material for: Mycotoxin Occurrence and Risk Assessment in Gluten-Free Pasta through UHPLC-Q-Exactive Orbitrap MS
Source: Toxins (Basel). 2021 Apr 25;13(5):305. doi: 10.3390/toxins13050305 (PMC8146712; doi:10.3390/toxins13050305)
Supplement: Supplementary file 1 [file toxins-13-00305-s001.zip › toxins-1184141-supp.pdf]

Supplementary Materials: Mycotoxin Occurrence and Risk Assessment in Gluten-free Pasta through UHPLC-Q-Exactive Orbitrap MS

Josefa Tolosa, Yelko Rodríguez-Carrasco, Giulia Graziani, Anna Gaspari, Emilia Ferrer, Jordi Mañes and Alberto Ritieni

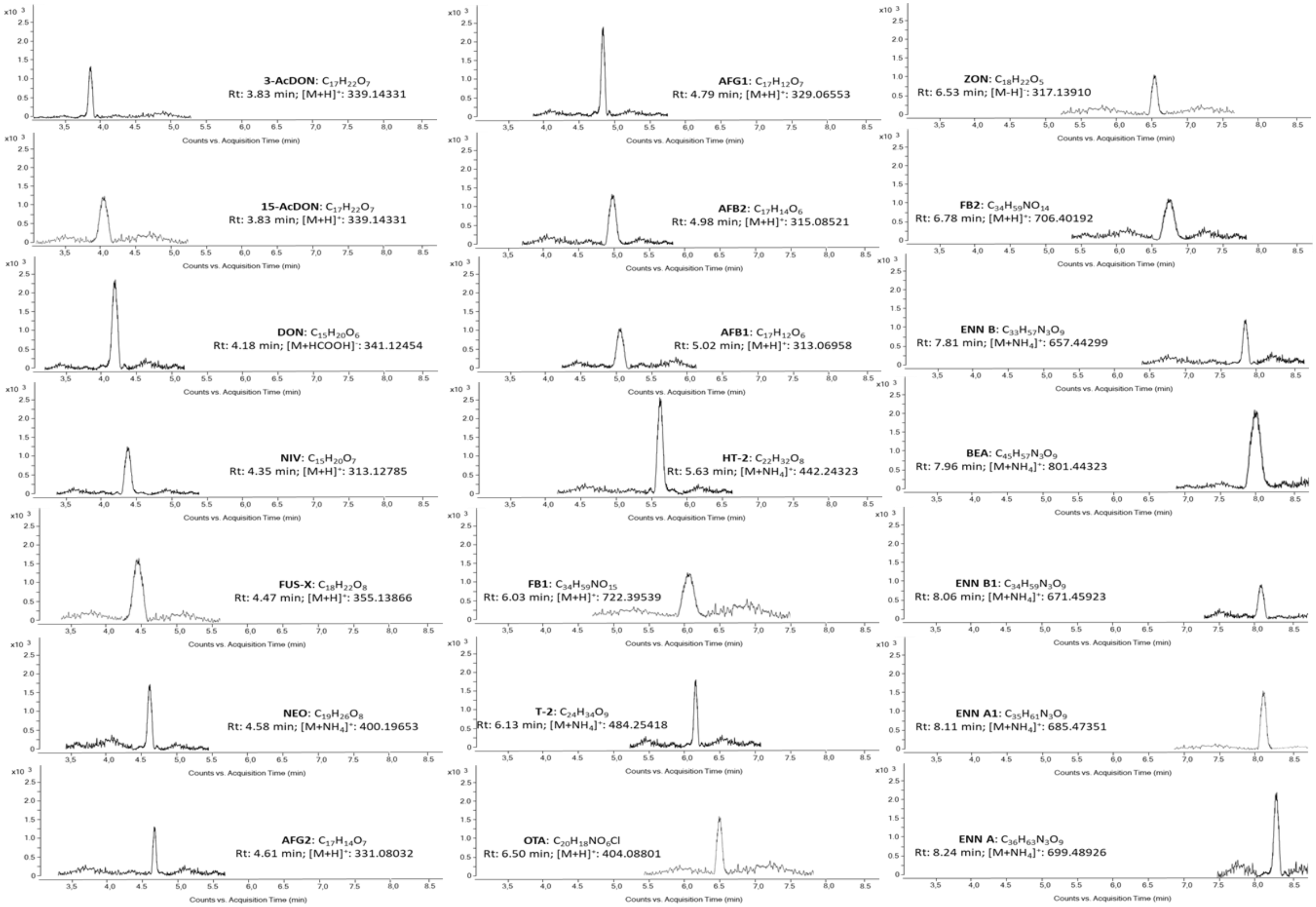

Figure S1. UPLC-Q-Orbitrap HRMS extracted ion chromatogram of a blank GF sample spiked at 12.5 μg/kg of each mycotoxin.
